# Pleuro-Pulmonary Extramedullary Plasmacytomas in Multiple Myeloma: A 15-Year Experience from a Tertiary Center

**DOI:** 10.3390/cancers18010019

**Published:** 2025-12-20

**Authors:** Sorina Badelita, Sinziana Barbu, Camelia Dobrea, Cerasela Jardan, Monica Popescu, Codruta Delia Popa, Claudia Toma, Larisa Zidaru, Mihai Emanuel Himcinschi, Horia Mihail Sandu, Didona Vasilache, Adelina Vlad, Daniel Coriu

**Affiliations:** 1 Clinical Department I, Hematology, Fundeni Clinical Institute, 022328 Bucharest, Romania; sorinabadelita@gmail.com (S.B.); daniel.coriu@umfcd.ro (D.C.); 2 Hematology Department, “Carol Davila” University of Medicine and Pharmacy, 050474 Bucharest, Romania; mhimcinschi@gmail.com; 3 Clinical Pneumology Department, “Dr. Marius Nasta” Hospital, 050159 Bucharest, Romania; 4 Biochemistry Department, “Victor Babes” University of Medicine and Pharmacy, 300041 Timisoara, Romania; 5 Physiology Department, “Carol Davila” University of Medicine and Pharmacy, 050474 Bucharest, Romania

**Keywords:** multiple myeloma, extramedullary disease, pleuro-pulmonary involvement, myelomatous pleural effusion, prognostic factors, progression-free survival

## Abstract

**Background/Objectives**: Extramedullary involvement in multiple myeloma represents an aggressive disease phenotype, associated with reduced survival and an unfavorable prognosis. Thoracic manifestations are rare and remain poorly characterized in the literature. **Methods**: We conducted a retrospective, single-center study at the Fundeni Clinical Institute, including patients diagnosed with multiple myeloma between February 2010 and February 2025. The study cohort consisted of 34 patients with infiltration of the pulmonary parenchyma, pleura, or the presence of myelomatous pleural effusion. Diagnosis was confirmed using a combination of imaging modalities (computed tomography or magnetic resonance imaging), cytological examination, immunophenotyping, and histopathological confirmation whenever feasible. **Results**: Out of a total of 2012 patients with multiple myeloma, the incidence of pleuro-pulmonary extramedullary involvement was 1.6%. The median age at diagnosis was 58 years. Pleuro-pulmonary disease was present at initial diagnosis in 26.5% of cases, while 73.5% developed it at relapse. The most common presentation involved combined pleural involvement and myelomatous effusion (70.6%). Adverse prognostic markers included elevated β2-microglobulin levels (in over 80% of cases) and increased lactate dehydrogenase (LDH) in approximately 50%. Cytogenetic abnormalities such as del(17p), t(4;14), t(14;16), t(11;14), and 1q gain were identified. The median overall survival (OS) from the diagnosis of pleuro-pulmonary extramedullary disease was 16 months, with a 2-year survival rate of 25%. No patient survived beyond 5 years. The median progression-free survival (PFS) was 9 months. **Conclusions**: Our findings confirm the aggressive clinical course and poor prognosis of these disease manifestations, mainly when they occur at relapse. In the absence of standardized treatment guidelines, individualizing therapy and accessing novel strategies may be essential for improving patient survival.

## Simple Summary

Patients with multiple myeloma can develop lesions outside the bone marrow, in the lungs and pleura, a rare but very aggressive form that has been little described so far. We retrospectively analyzed, at a single center, all cases diagnosed between 2010 and 2025 and identified 34 patients with pleuropulmonary involvement. We described how the disease occurs (at onset or relapse), how it manifests (pulmonary infiltrates, pleural involvement, pleural effusion), the diagnostic methods used, and survival. The results show an unfavorable evolution, with a median survival of 16 months and only a quarter of patients alive at 2 years. This study provides one of the largest series published on this topic and emphasizes the need for personalized treatments and modern strategies (e.g., targeted antibodies or modified T cells), providing the basis for future recommendations and clinical trials.

## 1. Introduction

Multiple myeloma (MM) is a relatively uncommon neoplasm, accounting for about 1% of all malignancies and just over 10% of hematologic cancers [1]. The disease may stay confined to the bone marrow or, less frequently, involve extramedullary sites. The ability of a malignant clone to proliferate and expand independently of the bone marrow microenvironment leads to the development of extramedullary plasmacytomas. Multiple myeloma patients may develop extramedullary disease due to various factors. The timing of EMD diagnosis is an important factor influencing the clinical course. Studies show that extramedullary disease is present at diagnosis in 6% to 20% of patients. The risk of developing extramedullary disease becomes higher at relapse, with reported incidence rates ranging from 13% to 26% during this period. EMD incidence rates during this period range between 13% and 26% [2]. Alkan O et al. reported an incidence of up to 18% [3], which seems to be more common in advanced disease stages or at relapse [4]. A possible hypothesis, also discussed by Bansal et al., suggests that the rising incidence of EMD in recent years may be partly related to the longer overall survival of MM patients due to increased access to modern therapies. As overall survival improves, the selective pressure may favor the emergence of more aggressive clones, ultimately resulting in extramedullary dissemination [5].

The most common sites of EMD include the skin, liver, kidney, and central nervous system (CNS) [6]. Myelomatous involvement of the pulmonary parenchyma or pleura is extremely rare. Patients with myelomatous pleural effusion account for less than 1% of cases [7], with higher prevalence in relapsed or refractory disease [6] and in the IgA MM subtype [3]. Despite intensive treatment, EMDP exhibits a progression-free survival and overall survival of less than 4 months [8].

Taken together, EMD is linked to a poor prognosis and significantly decreased overall survival. The presence of EMD in relapsed disease is associated with a median survival of only 6 months [5]. Additionally, myelomatous pleural effusion is regarded as an independent adverse prognostic factor [7], often linked with high tumour burden (indicated by elevated β2-microglobulin levels) and extensive involvement [7,9]; Median survival in this situation has been reported to be as short as 4 months [4].

Cutting-edge strategies, such as CAR-T cell therapies or B-cell maturation antigen (BCMA)-directed antibodies, have shown inferior responses in this patient population [10]. The Mayo Clinic guidelines, updated in 2021, recommend VTD-PACE–based regimens followed by consolidation with autologous stem cell transplantation (ASCT) in eligible patients [11]. More recent literature has reported encouraging outcomes with regimens incorporating next-generation immunomodulatory drugs (IMiDs), monoclonal antibodies, or proteasome inhibitors (PIs); however, the lack of prospective studies and the rarity of cases hinder the formulation of evidence-based therapeutic standards [11].

Current diagnostic strategies vary between institutions and may include computed tomography (CT), magnetic resonance imaging (MRI), positron emission tomography–computed tomography (PET-CT), as well as biopsies performed via bronchoscopy, surgical procedures, or percutaneous techniques.

A key example of how imaging methods could guide the diagnosis is integrating thoracic ultrasound into the initial assessment protocol for such patients. Its use is as an early diagnostic tool for detecting minimal pleural fluid that could be missed by conventional radiological investigations.

Currently, no consensus exists regarding the optimal treatment strategy for EMDP in MM. Newer agents, such as next-generation CELMoDs, have been mentioned as potential options, but the data supporting their use are still very limited. Mezigdomide has shown stronger activity than traditional immunomodulators in early laboratory studies [12], and some reports have raised the possibility that it might help in difficult cases, including those with pleural or pulmonary involvement. Even so, the situation remains uncertain, and much more clinical experience is needed before we can judge how useful these therapies might be in EMDP.

The limited availability of data on relapse and survival, along with the lack of standardized follow-up protocols, hampers the accurate assessment of risk and prognostic factors in EMDP associated with MM. There is therefore a clear need for prospective, multicenter, large-scale cohort studies to establish standardized protocols that could optimize both prognosis and survival in this patient population.

The present study was designed with two main objectives: to evaluate progression-free survival (PFS) and overall survival (OS) in patients with EMDP, to describe the clinical, biological, and imaging characteristics of these patients, and to investigate potential prognostic factors, thereby contributing to the expansion of this medical field.

Although pleuropulmonary involvement in EMD has been noted in case reports and a handful of small retrospective studies, current data remain scarce and inconsistent, failing to provide sufficient clarity for firm clinical guidelines. Numerous aspects remain uncertain: cytological, imaging, and immunophenotypic findings are rarely analyzed collectively; survival outcomes are poorly documented; and the true incidence of this manifestation is not well established. Given the rarity of this disorder, our cohort is among the largest to date, providing a more robust basis for evaluating endpoints such as PFS and OS. Our method of diagnosis introduces a method distinct from those previously reported, bringing to light a newly documented approach that differs from cited ones, enabling a more detailed understanding of disease manifestation and potentially facilitating earlier detection of aggressive extramedullary progression. Thus, our research provides important clinical insights in an area where standardized guidelines are still evolving.

## 2. Materials and Methods

### 2.1. Definitions

The diagnostic and response criteria for MM were enforced in accordance with the International Myeloma Working Group (IMWG) propositions that were valid at the time of treatment. EMD was defined as the presence of clonal plasma cell infiltrates in soft tissues, regardless of the axial skeleton. Plasmacytomas resulting from direct extension of adjacent bone lesions were excluded.

EMD manifestations in the pleuro-pulmonary region (EMDP) were defined as the presence of a mass or infiltration in the pleura or pulmonary parenchyma, or the occurrence of myelomatous pleural effusion, all confirmed by imaging studies. Depending on the clinical context, confirmation was obtained by cytological, immunophenotypic, or histopathological analysis. As a result, only clonal plasma cell accumulations affecting pulmonary parenchyma, pleura, or pleural fluid were considered as EMDPs of MM.

### 2.2. Study Design and Patients

This was a retrospective, single-center study conducted over a 15-year period (February 2010–February 2025), assessing patients diagnosed with MM to identify cases with EMDPs. Patients meeting these criteria were subsequently analyzed regarding their clinical profiles, diagnostic and therapeutic approaches, as well as survival outcomes.

Patient selection was based on the following inclusion criteria: a confirmed diagnosis of MM and documented evidence of EMDP established through imaging, pleural fluid analysis, or histopathological examination. The exclusion criteria did not play an important role, as we organized this study as an intention-to-treat type, which means that all patients who had a confirmed diagnosis of MM with any/or EMDP manifestations were included in the present article.

### 2.3. Data Collection and Diagnosis Process

Patient data were obtained from the National Romanian MM Registry, selecting only those cases treated at the Department of Hematology, Fundeni Clinical Institute. Imaging assessment included, as appropriate, CT, MRI, or PET-CT. Patients were diagnosed from 2010, a period of variable access to PET-CT and MRI in Romania; all underwent at least one imaging evaluation. Additionally, demographic variables, disease stage according to the International Staging System (ISS), the presence of cytogenetic abnormalities, therapeutic regimens administered, and treatment response were recorded.

From a radiological perspective, parenchymal lesions typically appear as tumor-like masses, while pleural involvement exhibits heterogeneous features, including nodular or infiltrative (thickening) patterns, as well as pleural effusion.

The pleural effusion fluid was collected in a Transfix^®^ tube (TF-CSF-S-10-RUO) for biological liquids. The BD OneFlow™ LST (Catalogue No. 658619, Becton Dickinson, San Diego, CA, USA) protocol was used for the pre-analytical steps of the preparation process, and the staining was strictly for surface membrane antigens (no cytoplasmic markers). The acquisition was performed using a BD FACSLyric (BD Biosciences, San Jose, CA, USA). The FCS 3.0 files obtained were further analyzed using Infinicyt Software, version 2 (Cytognos, Salamanca, Spain).

Pleural fluid cytology was performed using cytospin preparations and May–Grünwald Giemsa staining, revealing atypical plasma cells with prominent nucleoli and condensed or dispersed chromatin [4]. Flow cytometry immunophenotyping was also performed to establish a definitive diagnosis and exclude alternative conditions, analyzing markers including kappa, lambda, CD45, CD38, CD138, CD19, CD56, CD27, CD117, and CD81.

Electrophoresis and immunofixation of pleural fluid, performed in parallel with serum and urine analyses, demonstrated the same type of monoclonal protein. In patients with exclusively pleural or pulmonary involvement, histopathological examination was performed on the tumor formation (mass, nodule, or pleural thickening).

**Figure 1:**
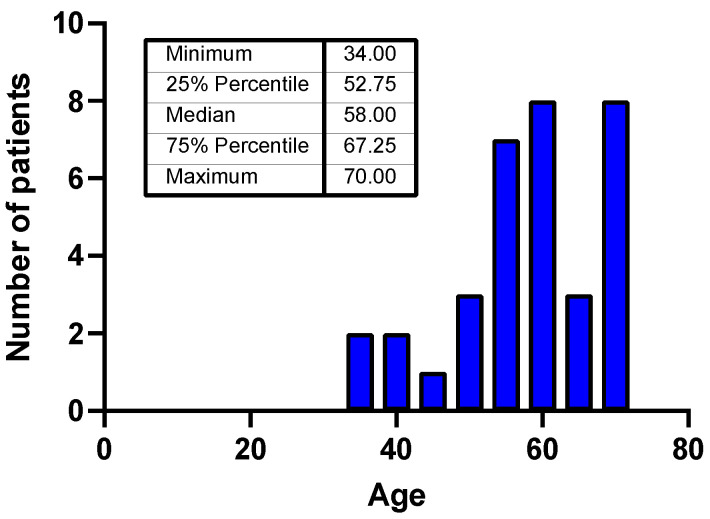
Age distribution of the patients included in the study.

**Figure 2:**
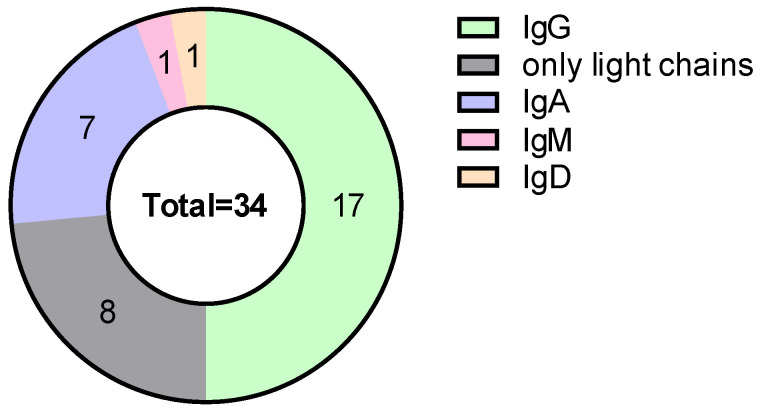
Distribution of monoclonal protein type.

**Figure 3:**
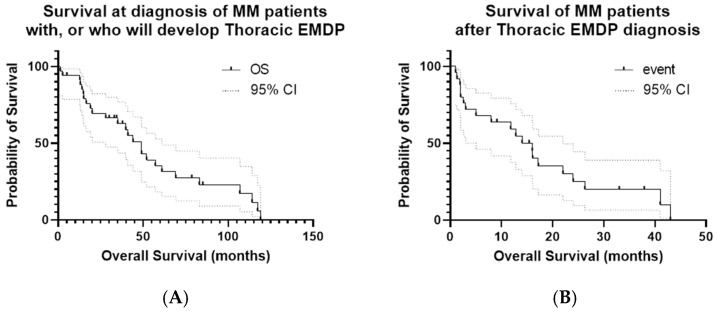

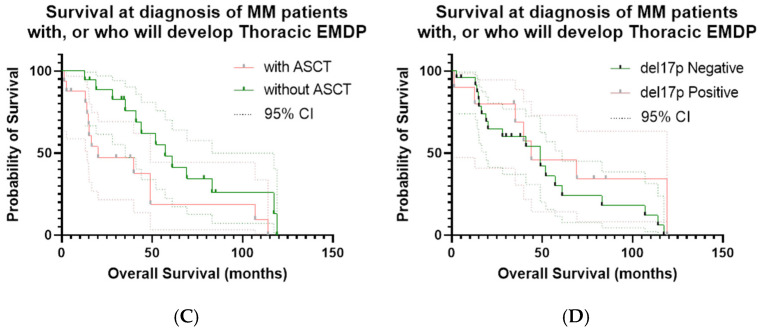
Kaplan–Meier curves for OS and PFS in the study cohort. (**A**) OS calculated from the date of initial MM diagnosis; event = death; OS from the time of EMDP diagnosis is also shown. (**B**) PFS calculated from the date of first EMDP documentation; event = disease progression or death. Median PFS: 9 months; 1-year PFS: 26.3%; maximum PFS: 25 months. (**C**) ASCT: patients who received ASCT versus those who did not; event = death; time zero = date of initial MM diagnosis. (**D**) OS according to del17p status: patients with del17p positivity versus those without; event = death; time zero = the date of initial MM diagnosis.

Tissue samples for pleuro-pulmonary lesions (2.5 μm sections) were stained with Hematoxylin and Eosin (HE) and Congo Red (Congo Red staining kit, with Ventana BenchMark special stain automat/Roche Diagnostic). For all cases with pathological/clinical suspicion of plasma cell proliferation on HE stain (Figure 1), automated immunohistochemistry (IHC) was performed (Ventana BenchMark Ultra, Roche Diagnostic), with antibodies against CD138 (clone B-A38 RTU, Cell Marque) (Figure 2), k light chain (Rabbit polyclonal primary antibody, RTU, Roche Diagnostic) and lambda light chain (Rabbit Polyclonal primary antibody, RTU, Roche Diagnostic) for clonality (Figure 3); CD20 (clone L26, RTU Roche) and CD56 (clone MRQ-42, Cell marque). Detection used an HRP polymerase system with DAB chromogen, enabling assessment of CD138, CD20, and CD56 in plasma cell infiltrates and light chain expression and restriction in plasma cells.

The differential diagnosis considered other potential solid or hematologic malignancies, infectious causes of pleural effusion, as well as complications of systemic diseases.

### 2.4. Treatment

Treatment selection was based on the therapeutic standards available at the time, while also considering drug availability in Romania. Eligible patients underwent autologous stem cell transplantation. The study cohort received combinations of immunomodulatory drugs (IMiDs), chemotherapy, proteasome inhibitors (PIs), and monoclonal antibodies, while three patients were treated with anti-BCMA–based therapy.

### 2.5. Statistical Analysis

Descriptive statistics were used to summarize patient and disease characteristics. Continuous variables were expressed as median (range) and categorical variables as frequencies and percentages.

Survival outcomes (PFS and OS) were estimated using Kaplan–Meier survival curves, which were compared using the log-rank test. Confidence intervals of 95% for the survival fractions were calculated using GraphPad Prism 9.3.0. OS was calculated from the time of MM diagnosis—time zero was the date of the initial MM diagnosis, and the curve describes the entire evolution of patients who subsequently developed EMDP. PFS was calculated from the time of thoracic EMDP diagnosis—time zero was the date of the first imaging/cytological/histological documentation of pleuropulmonary EMD; this analysis strictly reflects the prognosis after the occurrence of EMD. Kaplan–Meier test was also used to differentiate the survival outcomes when the entire cohort was stratified according to the therapeutic approach (with or without ASCT) or with the cytogenetic risk factor (with or without del17p+).

*p*-Values were considered statistically significant if *p* < 0.05.

### 2.6. Ethics Statement

The study was conducted in accordance with the principles of the Declaration of Helsinki. All data were collected retrospectively with the approval of the Ethics Committee of the Fundeni Clinical Institute (approval no. 36113/29.08.2025). Written informed consent for the use of medical data in research was waived by all patients at admission, in accordance with the organizational procedures of our university hospital.

## 3. Results

### 3.1. Baseline Characteristics

The study analyzed a cohort of 2012 patients successfully diagnosed with MM over a 15-year period (2010–2025) at the Department of Hematology, Fundeni Clinical Institute (patient characteristics are shown in Table 1). Patients with EMD, either at diagnosis or during the course of the disease, were identified. Extramedullary localizations were heterogeneous and included liver, stomach, pancreas, breast, subcutaneous tissue, nasopharynx, larynx, central nervous system (CNS), muscle, and lymph nodes. The present analysis included 34 patients who presented with pulmonary or pleural involvement or pleural effusion containing plasma cells.

In the study cohort, patient age at diagnosis ranged from 34 to 70 years, with a median of 58 years. Only one-third of patients were older than 65 years at diagnosis. The sex distribution was balanced, with 17 males and 17 females (Figure 1).

According to the type of monoclonal protein, 50% of patients had IgG secretion, 23.5% had light-chain MM, and 20.6% had IgA secretion. Light-chain restriction was balanced, with 50% kappa and 50% lambda (Figure 2).

At the time of MM diagnosis, most patients were classified as ISS stage I. At the detection of EMD, a relatively high frequency of adverse prognostic markers was observed. At the moment of relapse, over 80% of patients had β2-microglobulin serum levels exceeding 3.5 mg/L, and more than 60% had values greater than 5.5 mg/L, corresponding to ISS stage III. Elevated LDH levels above the upper limit of normal were documented in approximately 50% of patients assessed at relapse.

Fluorescence in situ hybridization (FISH) analysis detected del(17p) in 10 patients. In one case, this abnormality was linked with concurrent rearrangements of the immunoglobulin heavy chain (IGH) locus, specifically t(14;16) and t(11;14). Additionally, one patient showed an isolated t(4;14) translocation, while another had a 1q gain.

### 3.2. Clinical and Imaging Features of Pleuro-Pulmonary Involvement

Clinical manifestations were relatively widespread, including symptoms typically related to respiratory conditions such as perpetual cough, dyspnea, or chest pain. Imaging evaluation showed abnormalities like tumor-like masses, pleural thickening, or pleural effusion. Tumor masses were most often described as homogeneous soft-tissue lesions, without necrotic areas or calcifications.

MRI and PET-CT also proved useful for a more detailed characterization of the lesions, allowing assessment of tumor extension and its relationship with adjacent structures. In addition, PET-CT was employed as a tool for the detection of measurable residual disease (MRD) [13].

At the time of MM diagnosis, 26.5% of patients already exhibited pleuro-pulmonary involvement, whereas the majority (73.5%) developed this manifestation at relapse. The most frequent localization was myelomatous pleural effusion, documented in 70.6% of patients. Isolated pleural presentations, predominantly nodular lesions, were rare (5.9%). One female patient presented with concurrent multiple sites of disease, including pleural, pulmonary, and pleural effusion involvement, while 20.5% of patients had pulmonary parenchymal infiltration.

### 3.3. Treatment

Among the 34 patients, 25 (73.52%) experienced EMDP at relapse. Of these, 17 (50%) were heavily pretreated, having been exposed to multiple therapeutic classes. Disease progression with the appearance of this new type of involvement occurred during the second line of therapy in 10 (29.2%) of cases and after the third and fourth lines in 25% of cases each.

By the time EMDP was diagnosed, all patients had been exposed to proteasome inhibitors (PIs) and chemotherapy, and most had also received immunomodulatory drugs (IMiDs) and anti-CD38 monoclonal antibodies. Autologous stem cell transplantation was performed in 52.9% of patients during first-line therapy and in 14.7% at relapse.

Three patients had received bispecific antibody therapy for relapsed/refractory MM: one with an anti-GPRC5D agent and two with anti-BCMA antibodies. One patient treated with an anti-BCMA antibody achieved complete remission. The CR lasted for 12 months, after which the patient died from infectious complications. The patient who received anti-GPRC5D antibodies achieved only a minimal response lasting for 5 months. It is important to mention that he developed EMDP during anti-BCMA therapy. He had been exposed to multiple prior therapies over a 6-year progression of myeloma.

Local radiotherapy for pleural plasmacytomas was administered in only two patients. Radiotherapy remains a therapeutic tool in the management of EMD, providing local disease control and pain relief [11]. In the setting of high tumor burden, radiotherapy may also be employed as a bridging strategy before CAR-T therapy, offering not only local effects but also a systemic immunomodulatory impact [14].

Given the lack of a standardized treatment protocol for patients with EMD, therapeutic approaches varied. The choice of regimen was mainly influenced by drug availability and combinations in Romania at the time, as well as by international guidelines. Consequently, patients in the study received different combinations of immunomodulatory drugs, chemotherapy, proteasome inhibitors, and monoclonal antibodies (RAD-3, DRd-2, CyBorD-2, KRd-2, PACE-3, Teclistamab-2, MP-1, POMVd-1, PAD-4, DKd-1, Dexamethasone-1, Pd-1, Kd-1, LenDex-1, Talquestamab-1, CHOP-1, RAD+Bortezomib-1, Dara VTD-2, CHEP-1, VTD-1, CTD-1, VRD PACE-1). Owing to the marked heterogeneity of treatments and the limited number of patients within each therapeutic category, outcomes were not stratified by treatment modality, and no comparative assessment of therapeutic efficacy was performed.

Additionally, three patients were treated with bispecific antibodies (talquetamab and teclistamab). Observed responses to bispecific antibody therapy, based on this very limited number of patients (including one complete response), should therefore be considered preliminary, observational, and hypothesis-generating, and are not intended to support generalizable conclusions. Radiotherapy was used in two patients as a bridging strategy; any observed clinical responses in this context are purely observational and should not be interpreted as evidence of therapeutic efficacy or generalizability.

### 3.4. Survival Analysis (PFS and OS)

In the Kaplan–Meier analysis of patients with EMDP, the median overall survival (OS) was 49 months, with a 1-year survival rate of 94%, but a significant decline at 5 years (35%). By contrast, OS calculated from the time of thoracic EMD diagnosis was considerably shorter, with a median of 16 months, a 1-year survival rate of 59%, a 2-year survival rate of 25%, and 0% at 5 years, reaffirming the adverse prognostic impact of EMDPs compared with OS from the time of MM diagnosis. Progression-free survival calculated from the time of EMDP diagnosis had a median of 9 months, a 1-year PFS rate of 26.3%, and a maximum of 25 months, highlighting the refractory and progressive nature of the disease in this setting (Figure 3A,B). OS was also compared between patients who underwent stem cell transplantation (median 57.1 months) and those who did not (median 20 months), with a statistically significant difference (*p* = 0.032). Additionally, OS was analyzed according to del17p status: patients with del17p positivity had a median survival of 44 months, compared with 49 months for those who tested negative (*p* = 0.27, not statistically significant), consistent with the known unfavorable prognostic effect of del17p. These results are presented in Figure 3C,D.

## 4. Discussion

EMD in MM is widely recognized as an aggressive disease phenotype, driven by the ability of clonal plasma cells to adapt and survive outside the bone marrow microenvironment. A plasmacytoma is defined as a localized tumor lesion, either in bone (osseous plasmacytoma) or in soft tissues (extramedullary plasmacytoma). Three distinct mechanisms underlying the development of extraosseous plasmacytomas have been described in the literature: direct invasion of neighboring soft tissues due to cortical bone destruction by the tumor mass; hematogenous dissemination of plasma cells to organs or soft tissues without bone continuity; and, rarely, cases initiated by invasive medical procedures [6].

In 2021, the International Myeloma Working Group (IMWG) issued the latest classification of plasmacytomas, explicitly differentiating paraskeletal plasmacytomas, extramedullary plasmacytomas, and osseous plasmacytomas [15]. This classification is relevant to our analysis, as the definition used for EMD directly affects both its reported incidence and its prognostic implications.

Nevertheless, debate persists in the international literature about whether the direct extension of bone lesions (paraskeletal plasmacytomas) should be included in the definition of EMD. In our study, we included only myelomatous infiltrates confined to soft tissues. This approach enabled a more accurate comparison of our data with previously published case series. By excluding paraskeletal plasmacytomas, we aimed to minimize the risk of overestimating EMD incidence.

An essential point of comparison is the timing of EMD occurrences. Research findings from recent studies indicate that extramedullary disease appears more often during relapse than at diagnosis. The incidence rates of contemporary patient groups vary based on the research approach; one study indicates that extramedullary tumors occur in 7–18% of patients at their initial diagnosis and increase to 70% during relapse or later stages [16], while another study reports a diagnosis rate of 3–5% compared to a relapse rate of 6–20% [17].

The Balkan Myeloma Study Group reported significantly poorer survival outcomes for patients who developed extramedullary disease at the time of relapse, with a progression-free survival (PFS) of 9.1 months and an overall survival (OS) of 11.4 months [18].

With regard to pleuro-pulmonary involvement, there are no/too few available data on PFS, and information on overall survival is scarce. The reported OS is extremely poor, around 4 months [4], with more favorable outcomes described only in small series, such as the study by Gao et al., which reported an OS of 13 months in 13 patients with myelomatous pleural effusion [7].

Our results are consistent with prior data: 73.5% of patients in our cohort developed EMD at relapse, with a median overall survival (OS) of 16 months and a median progression-free survival (PFS) of 9 months. The explanation for our results is that our cohort is not entirely dominated by myelomatous pleural effusion (prognosis of 4 months), but also includes parenchymal/pleural infiltrates, and approximately 25% had EMD at MM onset. Patients were younger (median of 58 years) and therefore eligible for intensive therapies, with wide access to PI/IMiD/anti-CD38 and ASCT in the first lines. Multimodal diagnosis in a tertiary center allowed rapid intervention, and the recent period (2010–2025) corroborated with the strategies applied, explaining the advantage over series focused on relapse or dominated by MPE. Although the OS observed in our study was slightly higher than that reported by the Balkan Group, survival curves confirmed the same unfavorable trend for cases diagnosed at a later stage. Thus, our analysis supports the hypothesis that the timing of EMD occurrence is a major determinant of prognosis and that inclusion of this variable in risk stratification scores could enhance their predictive value.

Research data on EMDP remain scarce due to insufficient reporting on transplant status and high-risk cytogenetic markers. Most available studies consist of individual case reports or small series, which describe short survival durations but do not present Kaplan–Meier survival analyses stratified by autologous stem cell transplantation (ASCT) or by del(17p) status. Research on larger cohorts of MM patients outside the bone marrow shows that ASCT is associated with improved survival, but the study did not focus on thoracic MM cases. While studies in larger cohorts of patients with extramedullary multiple myeloma outside the bone marrow suggest an association between ASCT and improved survival, these analyses did not specifically focus on thoracic involvement [18,19]. n our study, patients who underwent ASCT had a median overall survival of 57.1 months, compared with 20 months for those who did not receive ASCT (*p* = 0.032), indicating a survival benefit associated with transplantation in this rare and clinically challenging condition. With respect to cytogenetic risk, patients with del(17p) had a median OS of 44 months, whereas those without del(17p) had a median OS of 49 months; this difference was not statistically significant. Although del(17p) is generally associated with inferior survival in MM, with reported OS of 47.3 months in previous studies [20], the lack of significance observed in our cohort may be attributable to the small sample size of our high-risk subgroups. Additional contributing factors may include differences in treatment exposure, timing of ASCT, and variability in the del(17p) cut-off used across studies. Overall, our findings provide novel survival data stratified by ASCT status and del(17p) in a well-defined cohort of patients with EMDP.

Another significant aspect of comparison relates to the demographic profile and type of paraprotein. Previous studies indicate a higher incidence of EMD in younger patients (<60 years) and in non-secretory or light chain–only myeloma subtypes [21]. In our cohort, the median age was 58 years, confirming this previously described trend and highlighting a profile of earlier disease onset with more aggressive behavior. Furthermore, 23.5% of patients presented with micromolecular myeloma, a proportion comparable to that reported in other series. These findings reinforce the association of non-secretory and light chain–only forms with an increased risk of EMD. Although differences did not reach statistical significance, the consistent direction with published data suggests that this factor warrants further investigation in larger multicenter studies.

Regarding serum biological markers, elevated LDH and β2-microglobulin levels have frequently been reported in patients with EMD [22].

In our series, more than 80% of patients had β2-microglobulin levels above 3.5 mg/L, and half presented with increased LDH levels, values that closely match those in the literature. This concordance further supports the idea that these two parameters remain simple, accessible, and clinically useful predictors for identifying patients at higher risk of developing EMD.

At the cytogenetic level, several studies have shown an association between EMD and high-risk abnormalities such as t(4;14), gain(1q21), and del(17p) [23]. In our cohort, FISH analysis displayed similar results: 10 patients had del(17p), including one with complex IGH rearrangements (t(14;16) and t(11;14)); one patient carried a t(4;14) translocation, and another showed 1q gain. Cytogenetics indicates poor prognosis in thoracic EMD because this entity is rich in high-risk FISH abnormalities, which mark aggressive biology and inferior outcomes. The prevalence of these lesions explains the reduced survival after the occurrence of pleuropulmonary EMD (median OS 16 months and PFS 9 months), confirming the unfavorable impact even in the era of modern therapies and thus justifying FISH testing for early identification and therapy adaptation. The agreement with the presented data highlights the gravity of FISH testing for early perceptions of high-risk patients and for balancing therapeutic strategies accordingly.

The literature further suggests that pleural plasmacytomas are noteworthy, making up about 3–6% of all EMD of MM [24,25]. In our cohort, the recurrence of these localizations was similar, with only two cases disclosed among the 34 patients analyzed (5.9%), thereby confirming the rarity of this presentation and the positioning of our findings next to previously published data.

Novel therapies might have an important, but more realistically modest role in EMDP. In general, both bispecific antibodies and CAR-T cell therapies demonstrate lower response rates in patients with myeloma without EMD manifestations [26]. This reduced efficacy is one of the reasons current guidelines and published series emphasize the need for dedicated clinical trials and the development of personalized treatment strategies, rather than establishing firm therapeutic standards at this time In the Fundeni cohort, three patients received bispecific antibodies (one anti-GPRC5D and two anti-BCMA), of whom one treated with anti-BCMA achieved complete remission. This observation suggests that a subset of patients may derive meaningful benefit from these therapies, even in the presence of an aggressive biological phenotype. In parallel, local radiotherapy may be considered as a “bridging” strategy prior to CAR-T therapy, providing locoregional disease control and potential immunomodulatory effects, particularly in cases with high tumor burden. Overall, our findings support the continued exploration of bispecific antibodies and CAR-T therapies, ideally within controlled clinical trials, as the current evidence remains limited and heterogeneous.

Taken together, this study presents one of the most comprehensive retrospective series of EMDP cases reported to date. Our results are largely consistent with the existing literature, confirming both the aggressive biological profile of EMD and the poor prognosis of patients in whom it emerges at relapse. Nevertheless, the median overall survival observed in our series was slightly better than that reported by other groups. This discrepancy may be explained by the characteristics of our cohort and possibly by the therapeutic strategies employed, but it also raises the hypothesis of specific biological or demographic factors within the studied population.

The main limitations of our study are the retrospective and single-center design, along with the small sample size, which limits the generalizability of the results. In addition, survival analyses were descriptive in nature; owing to the limited cohort size and number of events, no formal statistical testing or multivariable modeling was performed to assess the prognostic impact of clinical variables, including the timing of extramedullary disease occurrence at diagnosis versus relapse.

Another limitation might be represented by the difficulty of integrating a COX model with a time-dependent covariate for EMDP for our particular cohort, given the rather small number of participants and the difficulty of cohort matching in order to obtain a comparison “standard” group. Finally, the heterogeneity of our data, collected over a 15-year period, limits the feasibility of multivariable statistical analyses and the uniformisation of, in some cases (for example, imaging investigations), the data availability. However, they offer a solid foundation for future prospective multicenter studies. Combining our data with the existing literature underscores the importance of standardized diagnostic and monitoring protocols and the need to identify risk factors that enable early recognition of subgroups at higher risk of extramedullary progression/invasion.

## 5. Conclusions

The results of our study, based on the evaluation of 34 patients, show that EMDP in MM, although rarely reported in the literature, was present in 1.6% of patients in our cohort. EMDPs typically develop during the course of the disease, especially in patients with relapsed/refractory myeloma, often starting from the second line of treatment. The median for overall survival was, in appearance, unsatisfactory, at 16 months, with a 2-year survival rate of 25% and no survivors beyond 5 years, emphasizing the intricate, aggressive nature and adverse prognosis of this disease manifestation. These findings highlight the importance of early diagnosis and the need for personalized therapeutic strategies. In the absence of dedicated treatment guidelines for this rare entity, enrollment in clinical trials and access to innovative therapies such as bispecific antibodies, CAR-T cell therapies, or their combinations with conventional immunotherapy may offer viable options to improve survival and patient outcomes.

It should also be highlighted that, although infrequent, EMDP in MM can resemble primary lung cancer, pleural metastases, or tuberculosis. Pulmonologists are often the initial specialists who encounter the specified clinical cases, and their involvement is crucial for a prompt diagnosis. Recognizing certain aspects as a potential for differential diagnosis in cases of unexplained pleural effusion or pulmonary involvement can significantly impact patient management and prognosis.

## Figures and Tables

**Table 1:** Patient characteristics—This table summarizes the baseline demographic, clinical, and laboratory characteristics of the patients included in the study.

	N	Percent
Number of patients	34	100%
Sex		
M	17	50%
F	17	50%
Age at diagnosis ≥ 65 (years)	10	29.4%
Median Age at diagnosis (years)	58	
Type of response		
CR	2	6.25%
PR	10	31.25%
PD	7	21.87%
SD	5	15.62%
MR	1	3.125%
VGPR	7	21.87%
ISS staging		
Stage I	5	14.7%
Stage II	8	23.5%
Stage III	21	61.8%
Beta-2 Microglobulin > 3.5 (mg/L)	29	85.3%
Beta-2 Microglobulin > 5.5 (mg/L)	21	61.8%
LDH > 300 (U/L)	17	50%
EMDP site		
Pleura	2	5.9%
Lung	7	20.6%
Pleural fluid	24	70.6%
Pleura & Pleural fluid & Lung	1	2.9%
EMDP diagnosis		
At the time of MM diagnosis	9	26.5%
After MM diagnosis	25	73.5%

## Data Availability

All data concerning the publication of this article can be requested from the corresponding author.

## Abbreviations

The following abbreviations are used in this manuscript:

anti-BCMA	Antibody targeting B-cell maturation antigen
ASCT	Autologous Stem Cell Transplantation
CAR-T	Chimeric Antigen Receptor T-cell
CELMoDs	Cereblon E3 Ligase Modulators
CI	Confidence Interval
CNS	Central Nervous System
CR	Complete Response
CT	Computed Tomography
CyBorD	Cyclophosphamide + Bortezomib + Dexamethasone
DRd	Daratumumab + Lenalidomide + Dexamethasone
DVd	Daratumumab + Bortezomib + Dexamethasone
EMD	Extramedullary Disease
FISH	Fluorescence In Situ Hybridization
GPRC5D	G protein-coupled receptor, class C group 5 member D
IGH	Immunoglobulin Heavy Chain
IMiD	Immunomodulatory Drug
IMWG	International Myeloma Working Group
ISS	International Staging System
KRd	Carfilzomib + Lenalidomide + Dexamethasone
LDH	Lactate Dehydrogenase
MRI	Magnetic Resonance Imaging
MRD	Minimal Residual Disease
ORR	Overall Response Rate
OS	Overall Survival
PACE	Cisplatin + Doxorubicin + Cyclophosphamide + Etoposide
PET-CT	Positron Emission Tomography—Computed Tomography
PFS	Progression-Free Survival
PI	Proteasome Inhibitor
PVd	Pomalidomide + Bortezomib + Dexamethasone
RAD	Lenalidomide + Doxorubicin + Dexamethasone
VMP	Bortezomib+ Melphalan + Prednisone
VTD-PACE	Bortezomib + Thalidomide + Dexamethasone + Cisplatin + Doxorubicin + Cyclophosphamide + Etoposide
EMDP	EMD manifestations in the pleuro-pulmonary region
